# A Platinum and Porcelain Crown Found Useful in Certain Cases

**Published:** 1903-10

**Authors:** N. A. Stanley

**Affiliations:** New Bedford, Mass.


### A PLATINUM AND PORCELAIN CROWN FOUND USEFUL IN CERTAIN CASES.

BY N. A. STANLEY, D.M.D., NEW BEDFORD, MASS.

I do not claim anything new or original for the crown I wish to describe; in fact, most new things are but modifications of something already in use.

A form of fracture occasionally met with is where the buccal cusp of the bicuspid has broken off, the pulp undisturbed and in healthy condition. We do not feel warranted in devitalizing and setting an all porcelain crown, much less should we resort to the inartistic, labor-saving gold cap. The facings procured at the dental depots cannot be used in connection with the cap without encroaching upon the pulp, or being too prominent.

To obviate these difficulties I proceed as follows:

After preparing the tooth a band or tube of platinum, 32 or 33 gauge, is fitted. It is well to have this strip of platinum a little longer than the circumference of the tooth; for in making the joint I usually make a short cut in one end to a line denoting the exact circumference of the tooth, insert the other end of the band, both ends having been brought to a knife edge, and solder with pure gold. You now have a joint that cannot change, no matter to how high a temperature it may be subjected. With a pair of plate scissors and small stones the occlusion is quickly adjusted, which should not be too close, as we must allow for a thickness of solder on the occlusal surface.

Next cut out the buccal surface to a line corresponding to the fractured surface of the tooth, the edges of which should be dressed down all they will stand, particularly the meso-buccal, so that the adjoining teeth may hide, if possible, the line of union between platinum and porcelain.

Solder a piece of platinum to this surface, always using pure gold.

Up to this point the process is quite similar to that used in making the jacket crown described by Dr. Capon, of Toronto.

Next, a piece of platinum is soldered to the occlusal surface. It will be seen that it is well to have the part which is to protect the porcelain extend a little beyond the cusps of the adjoining teeth. This part of the platinum is now bent so as to correspond in length to the adjoining teeth, and can be reinforced if desired with a small piece of platinum. Now solder a small loop of platinum wire to the buccal surface which will be concealed by the porcelain and anchor the same.

The next step is to bake the porcelain to the buccal surface, and then cover the occlusal surface with solder, which is easily done in the Hammond furnace.

By watching carefully you can "sweat" a piece of soldier just where you want it. The overhanging edge of platinum has served to protect the porcelain from becoming chipped, and is now finished down and the crown gold-plated if desired. After the crown is ready for the porcelain I usually take an impression and then dismiss the patient, finishing at my leisure.

